# β-Carotene, a Potent Amyloid Aggregation Inhibitor, Promotes Disordered Aβ Fibrillar Structure

**DOI:** 10.3390/ijms24065175

**Published:** 2023-03-08

**Authors:** Siddhartha Banerjee, Divya Baghel, Ana Pacheco de Oliveira, Ayanjeet Ghosh

**Affiliations:** Department of Chemistry and Biochemistry, The University of Alabama, 1007E Shelby Hall, Tuscaloosa, AL 35487, USA

**Keywords:** β-carotene, amyloid aggregation, AFM-IR, fibril structure, beta-sheet

## Abstract

The aggregation of amyloid beta (Aβ) into fibrillar aggregates is a key feature of Alzheimer’s disease (AD) pathology. β-carotene and related compounds have been shown to associate with amyloid aggregates and have direct impact on the formation of amyloid fibrils. However, the precise effect of β-carotene on the structure of amyloid aggregates is not known, which poses a limitation towards developing it as a potential AD therapeutic. In this report, we use nanoscale AFM-IR spectroscopy to probe the structure of Aβ oligomers and fibrils at the single aggregate level and demonstrate that the main effect of β-carotene towards modulating Aβ aggregation is not to inhibit fibril formation but to alter the secondary structure of the fibrils and promote fibrils that lack the characteristic ordered beta structure.

## 1. Introduction

Alzheimer’s disease (AD) has been characterized as one of the most common causes of dementia. Approximately 6.5 million Americans age 65 and above are suffering from this disease, and this number is expected to more than double over the next few decades [1]. While the clinical symptoms of AD include cognitive decline and personality, mood, or behavioral changes [2,3], the pathological hallmark for the disease is the presence of extracellular plaques in the cerebral cortex which are mainly composed of misfolded aggregates of the amyloid β (Aβ) peptide [4,5]. Aβ peptides, having 39–43 residues, are generated from sequential enzymatic cleavage by β-secretase and γ-secretase [6,7]. The peptide with 42 residues (Aβ42) has been identified to aggregate faster in vitro compared to its 40 residue isoform, Aβ40, and the former have the most neurotoxic effects [8,9,10]. Although the precise correlation of plaques with neurodegeneration is yet to be fully understood [11], it has been demonstrated that amyloid aggregates play a crucial role in the development of AD. This process is accelerated by neuroinflammation and deposition of tau aggregates inside the neuron cells, leading to ultimate cell death [12,13]. Additionally, Aβ stimulates oxidative stress and further modulates AD progression [14,15]. Several therapeutic strategies have been applied to target Aβ aggregation, including inhibition of Aβ monomer aggregation, removal of amyloid aggregates, and immunotherapy [16,17,18]. However, despite significant efforts, the progress towards effective treatment of AD and development of therapeutics have been limited.

A number of natural products, including β-carotene, resveratrol, and epigallocatechin gallate are known to have a promising role in preventing several neurodegenerative diseases [19,20]. Lower levels of β-carotene in plasma have been shown to correlate with severity of AD patients compared to the control cases [21]. In addition, β-carotene has been found to inhibit the aggregation of Aβ [22] and assists in improving cognitive function [23]. More recently, β-carotene has been found to colocalize in amyloid plaques in AD diseased tissues [24,25], and while a possible anti neuroinflammatory role has been suggested, the exact reason for the presence of β-carotene in plaques is unclear. In vitro studies have demonstrated that β-carotene inhibits amyloid aggregation [26], leading to lower beta sheet concentrations in aggregation mixtures, which is typically used to gauge fibril formation. Although these studies clearly indicate the direct effect of β-carotene in modulating AD and amyloid aggregation, little is known about the molecular mechanism behind β-carotene induced amyloid inhibition. It should be noted that, in the general context of amyloid aggregation, inhibition usually implies prevention or significant reduction of fibrillation. Prior studies on the effect of β-carotene on amyloid aggregation have used thioflavin fluorescence as a spectroscopic marker of fibrils [26], wherein reduced fluorescence intensity in the presence of β-carotene was interpreted as reduced fibril formation. The aggregation kinetics have also been found to be slower, which is, again, assessed with fluorescence. The effect of β-carotene on the structure of amyloid aggregates, however, has not been investigated in detail. This gap in knowledge stems in part from the limitations of conventional biophysical techniques towards investigating the structure of individual aggregates. Without the ability to identify the individual members of the structural ensemble, it is difficult to unequivocally and fully understand the exact role of β-carotene in altering, and thus modulating, amyloid aggregation. In this report, we employ atomic force microscopy (AFM) augmented with infrared (IR) spectroscopy to understand structural changes to amyloid aggregates in presence of β-carotene. AFM-IR simultaneously offers nanoscale morphological characterization combined with aggregate-specific spectral and/or structural information. This allows for determination of the secondary structure of individual Aβ aggregates. It thus enables identification of the structural modulation that occurs due to β-carotene interaction with Aβ at the single aggregate level, which is difficult to follow by other techniques such as NMR and FTIR, which provide the average structural information of the ensemble.

Our findings demonstrate that β-carotene does not alter the morphology of Aβ42 aggregates, or inhibit fibril formation, therefore leading to markedly less abundance of fibrillar aggregates. However, it significantly changes the secondary structure of aggregates. In presence of β-carotene, amyloid fibrils adopt a secondary structure which lacks the expected highly ordered beta sheet structure, and, rather, contains predominantly non-beta sheet structural elements, whereas, in its absence, Aβ42 aggregates primarily adopt highly ordered beta sheet conformation.

## 2. Results and Discussion

**Figure 1:**
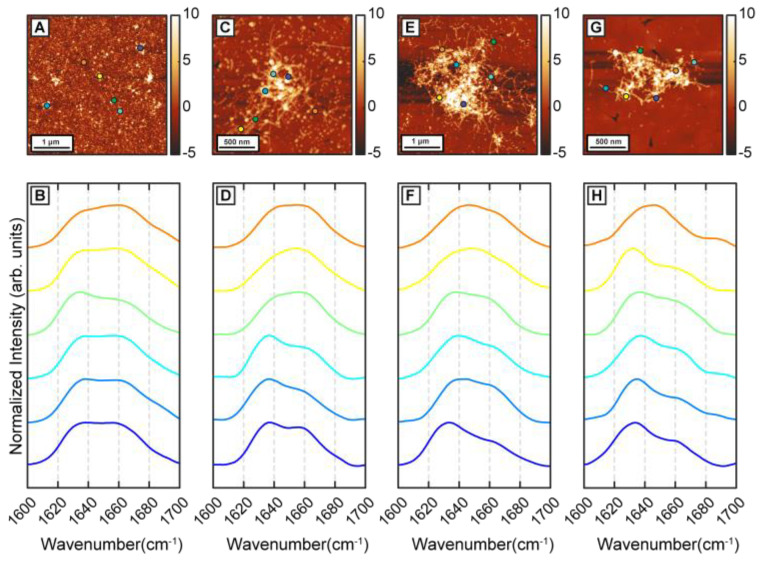
AFM-IR structural characterization of Aβ42 aggregates. (**A**) AFM topograph of Aβ42 oligomer formed after 1 h of incubation. (**B**) Representative IR spectra obtained from several oligomers. (**C**) AFM topograph of Aβ42 fibrils generated after 6 h and (**D**) representative IR spectra obtained from the sample. (**E**) AFM image of Aβ42 fibrils after 24 h and (**G**) 72 h. IR spectra obtained from several points on fibrils from (**F**) 24 h and (**H**) 72 h sample. Dots on the AFM topographs show the corresponding location of IR spectra.

To determine the effect of β-carotene on the structural transformation of Aβ42 aggregates, first we followed the structural evolution of Aβ42 through different stages of aggregation in a control experiment. Aggregation was performed at 37 °C in 50 mM 2-(N-morpholino)ethanesulfonic acid (MES) buffer, pH 6.5, with 100 mM NaCl and 0.5 mM EGTA without any external agitation. Aβ42 oligomers were observed in AFM topographs after 1 h of incubation (Figure 1A). Nanoscale IR spectra were obtained from individual oligomers to understand the distribution of their secondary structures (Figure 1B). It should be noted that the spectra shown are representative of the all spectra acquired and are not meant to be reflective of any underlying statistical distribution. The spatial locations corresponding to the IR spectra are shown with color-coded dots on AFM topographs. IR spectra show a peak at 1634 cm^−1^ with a shoulder at 1666 cm^−1^ (Figure 1B), consistent with presence of beta sheet structure. After 6 h of incubation, short fibrils are observed along with the oligomers (Figure 1C). IR spectra obtained from the fibrillar aggregates show similar features as seen in 1 h samples (Figure 1D). IR spectra are acquired from different points on the same fibril and from different fibrils and oligomers. The majority of the spectra have a peak at 1636 cm^−1^ with a shoulder at 1666 cm^−1^ and varying shoulder peak intensity (Figure 1D). The variation in spectra between different aggregates, i.e., the spectral heterogeneity is significantly larger in the 6 h sample compared to the oligomers observed after 1 h. After 24 h of incubation, denser fibrillar networks are observed (Figure 1E). IR spectra remain similar to the previous time points, having the peak at 1634 cm^−1^ with the shoulder at 1666 cm^−1^ (Figure 1F) and continue to exhibit variations between different fibrils. We note that, at these stages of aggregation, it is difficult to acquire spectra on an isolated or individual fibril, and the spectral measurements mostly correspond to fibrillar components that are a part of a larger network. Aggregation continued up to 72 h, where mainly fibrillar networks are present in the sample (Figure 1G). IR spectra are similar to the fibrils generated at earlier time points (Figure 1H): the majority of the spectra show a peak at 1634 cm^−1^ with a shoulder at 1664 cm^−1^ (Figure 1H). The amide I region of IR spectra is well known to reflect the secondary structure and has been extensively used to determine structural distributions of proteins and peptides, and of amyloid aggregates in particular. An amide I peak at ~1630 cm^−1^ has been shown to correspond to highly ordered β sheet structure, whereas a peak at ~1660–1670 cm^−1^ implies presence of non-beta-sheet structure, typically arising from a combination of disordered random coils and beta turn structural components [27,28]. For the Aβ42 aggregates studied in this report, we observe an amide I peak at 1634–1636 cm^−1^, demonstrating the presence of ordered beta structure. However, the amide I band also exhibits a shoulder at ~1666 cm^−1^, which indicates the presence of somewhat disordered structures that are most likely beta turn arrangements. Additionally, we observe spectral variations or heterogeneity at all stages of aggregation, including fibrils. Since the amide I spectra mirrors the secondary structure of the aggregates, spectral differences essentially imply structural variations. We have previously identified this unique structural aspect of Aβ, and demonstrated that early-stage fibrils are spectrally and, hence, structurally heterogeneous, which arises from heterogeneity in prefibrillar aggregates, such as oligomers and protofibrils. It is important to note in this context that the heterogeneity is not confined to different fibrils: different spatial locations of the same fibril can exhibit spectral differences, which we have previously reported [29]. In other words, the heterogeneity does not reflect the presence of different fibrillar or prefibrillar species with distinct structures, but rather represents structural heterogeneity within the same aggregate. The results reported here serve as a validation of structural heterogeneity in Aβ aggregates as a persistent feature that prevails under a range of aggregation conditions.

**Figure 2:**
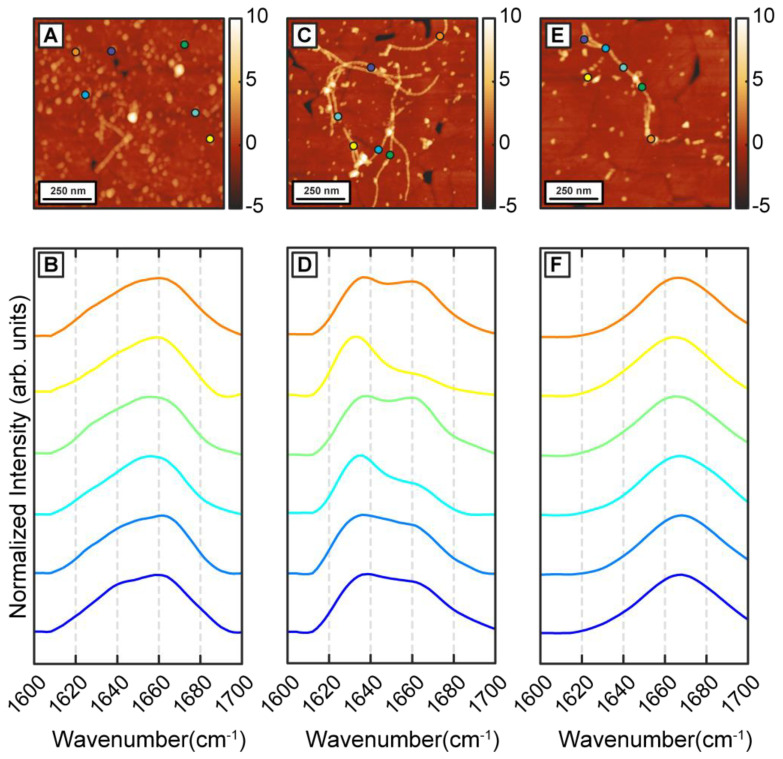
AFM-IR characterization of Aβ42 aggregates in presence of β-carotene (1:1 molar ratio) for 1 h and 6 h. (**A**) AFM topograph of Aβ42 oligomers formed after 1 h of incubation. (**B**) Representative IR spectra obtained from the oligomers. (**C**) AFM images for 6 h sample with the presence of one type of structural polymorph and (**D**) IR spectra obtained from it. (**E**) AFM topograph of other type of fibrillar structural polymorph obtained after 6 h and (**F**) representative IR spectra obtained from them. Dots on the AFM topographs show the corresponding location of IR spectra.

**Figure 3:**
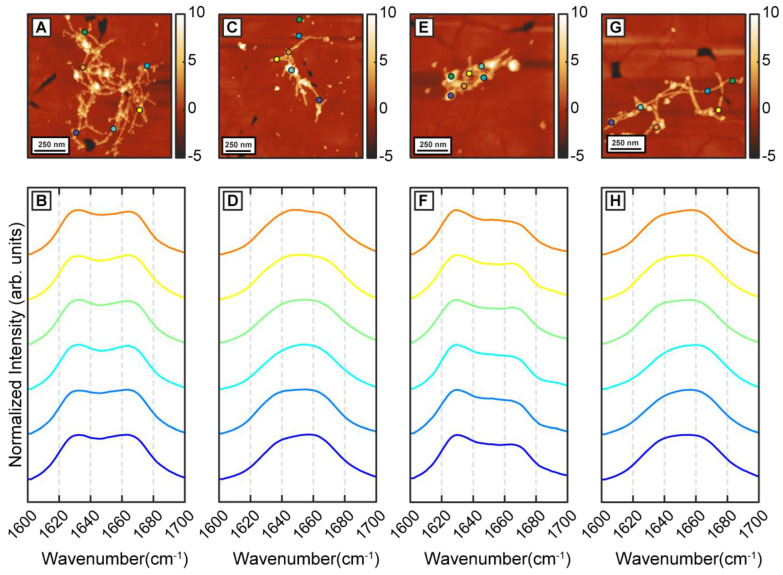
AFM-IR characterization of Aβ42 aggregates in presence of β-carotene (1:1 molar ratio) for 24 h and 72 h. (**A**) AFM topograph of one type of Aβ42 fibrillar structural polymorph obtained after 24 h and (**B**) representative IR spectra obtained from them. (**C**) AFM topograph of other type of structural polymorph obtained after 24 h and (**D**) representative IR spectra obtained from them. (**E**,**G**) AFM image of two types of fibrillar structural polymorphs obtained after 72 h and (**F**,**H**) their representative IR spectra. Dots on the AFM topographs show the corresponding location of IR spectra.

To understand how β-carotene may modulate the structure and ensemble of Aβ aggregates discussed above, we incubated Aβ in presence of equimolar β-carotene. This is in the range of relative concentrations that have been demonstrated to affect the aggregation of Aβ [26]. The aggregation condition, besides the addition of β-carotene, was kept similar to the control experiment. After 1 h of aggregation, primarily oligomeric species were observed in addition to a few small protofibrillar aggregates (Figure 2A). The spectra, shown in Figure 2B, exhibit a clear difference from the control measurements. The spectra exhibit a peak at 1654 cm^−1^ and a less intense shoulder at 1634 cm^−1^ which indicate a lack of well-defined beta sheet structure. One possible explanation for this is potential spectral interference from β-carotene, but this is unlikely because the absorption intensity of beta-carotene in the amide I region is significantly weaker (Figure S1). The background signal from pure β-carotene, particularly in AFM-IR, presents itself as a broad, featureless band that is also much weaker than the typical protein spectrum, thus ruling out possible distortion of the protein amide signal from carotenoid bands (Figure S2). It should be noted in this context that, in an AFM-IR experiment, we acquired a photothermal infrared signal, wherein the thermal expansion due to infrared absorption is measured instead of transmission of infrared radiation. Therefore, compared to bulk infrared spectra, the background signal from certain species can be different, depending on their thermal response. Moreover, the samples studied herein were rinsed prior to the AFM-IR measurements, which removed all/most of bulk β-carotene, so the chances of spectral interference were further minimized. The locations of the spectra on the AFM images are shown with color coded dots. In secondary structure analysis of proteins with infrared spectroscopy, a peak at 1654 cm^−1^ can be attributed to helical or random coil/disordered motifs; however, our measurements cannot unequivocally ascertain the exact origin of this peak. We note that while the majority of spectra exhibit a prominent peak at around 1654 cm^−1^, a few (~4% of all spectra acquired for 1 h aggregates) exhibit a pronounced peak at the typical beta sheet frequency (Figure S3). However, these spectra represent a very small fraction of the overall data, and it is unclear if these spectral variations are statistically significant. Taken together, the spectra indicate that the dominant secondary structural motif at this stage is non-beta-sheet in nature. The spectra from the protofibrillar species are similar to the oligomers. Representative protofibrillar spectra are shown in the Supporting Information (Figure S3). Interestingly, while spectra from individual oligomers bear signatures of a disordered structure, the ensemble is significantly more homogeneous as evidenced by the spectral distribution, and the spectral variations observed for pure Aβ aggregates are distinctly absent. After 6 h of aggregation, mostly fibrils are observed in the AFM measurements (Figure 2C,E). However, in contrast to pure Aβ fibrils, the fibrils can be categorized into two distinct subtypes based on their spectra, as shown in Figure 2D,F. One subtype exhibits an amide I peak at 1666 cm^−1^ with a less intense shoulder at 1634 cm^−1^, indicative of a primarily disordered structure (Figure 2F). While fibrils with disordered secondary structure may seem counterintuitive, tau fibrils have been shown to exhibit similar structure [30]. The other subtype shows a prominent peak at 1634 cm^−1^, suggesting a primarily beta sheet structure (Figure 2E). One key difference between this spectral variation and that observed in pure Aβ fibrils is that these spectra are fibril specific: in other words, different fibrils exhibit different spectra and therefore different structure. This is not entirely unexpected in the context of amyloid aggregation, and the presence of polymorphs in amyloid aggregate ensembles is well-known [31]. It has also been demonstrated that different polymorphs can differ with respect to the molecular arrangement of the beta strands [31]. However, we did not observe any significant morphological differences between the two fibril subtypes, which is typically the characteristic of polymorphism. We therefore conclude that these fibrils are not strictly polymorphs in the morphological sense but rather represent ‘structural polymorphs’: that is, they have different secondary structure but similar morphology. We have previously identified evidence of such structural polymorphism in fibrillar aggregates of tau [30] and short Aβ derived peptide sequences [32], but never in Aβ itself. Fibrils generated after 24 h and 72 h of aggregation exhibit the same trend (Figure 3A–H) In both cases, we identified the presence of two distinct fibril subtypes that exhibit the above spectral differences (Figure 3B,D,F,H). Interestingly, while the aggregation pathway now shows the presence of two distinct structural entities, the structural and/or spectral heterogeneity within these fibrillar subtypes is noticeably absent. The spectra from each subtype do not exhibit significant variations between themselves as was observed for pure Aβ fibrils. The above results, taken together, unequivocally demonstrate that β-carotene alters the aggregation pathway of Aβ instead to simply inhibiting the kinetics of aggregation. At equimolar concentration, β-carotene does not impede fibril formation; on the contrary, it acts to change the structural distribution of fibrils. Incorporation of β-carotene in the aggregation mixture leads to two major changes: firstly, the resultant fibrils exhibit structural polymorphism and can be categorized into two types that are either more beta-sheet-like or more disordered. Secondly, the structural sub ensemble corresponding to each polymorph is significantly more homogeneous than pure Aβ. These results can also be interpreted from a different perspective. Since the presence of beta sheet structure in mature Aβ fibrils is well known, and is also seen in our control measurements, it can be argued that the key structural change induced by β-carotene in fibrils is structural disorder. That is, β-carotene possibly leads to formation of fibril species that have primarily non-beta-sheet secondary structures, and thus lack the structural order present in stacked beta sheets.

**Figure 4:**
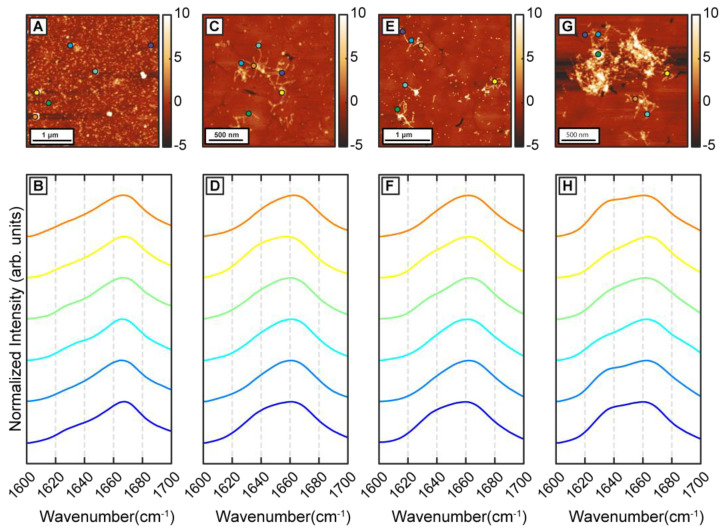
AFM-IR characterization of Aβ42 aggregates in presence of β-carotene (1:3 molar ratio). (**A**) AFM topograph of Aβ42 oligomer formed after 1 h of incubation. (**B**) Representative IR spectra obtained from oligomers. (**C**) AFM topograph of Aβ42 fibrils generated after 6 h and (**D**) representative IR spectra obtained from the sample. (**E**) AFM image of Aβ42 fibrils after 24 h and (**G**) 72 h. IR spectra obtained from several points on fibrils from (**F**) 24 h and (**H**) 72 h sample. Dots on the AFM topographs show the corresponding location of IR spectra.

**Figure 5:**
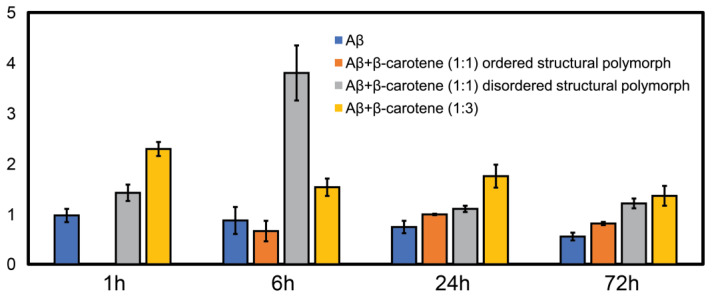
Ratio of intensity of amide I band at 1666 cm^−1^ to that at 1636 cm^−1^ at different time-points during aggregation. Higher value of ratio indicates relative increase in non-beta sheet/disordered structure. The data point for ordered polymorph at 1 h is missing, since no such aggregates were found. The ratios were calculated for each of the spectra shown in Figure 1, Figure 2, Figure 3 and Figure 4, and mean values are shown here. Error bars denote the standard deviation.

To better understand the precise effect of β-carotene on fibril structure, we aggregated Aβ in presence of threefold higher β-carotene concentration (1:3 molar ratio). If β-carotene indeed promotes structural disorder in fibrils, at higher molar ratios, the abundance of disordered fibril species is expected to increase. As before, we observed oligomers after 1 h of incubation (Figure 4A). IR spectra obtained from several individual oligomers showed homogeneous spectral distribution, having a peak at 1666 cm^−1^ with a weaker shoulder at 1628 cm^−1^ (Figure 4B). This is consistent with the aggregates formed in presence of equimolar β-carotene and would suggest a minimal impact of increased concentration of β-carotene on the aggregation pathway. The spectral spatial locations are shown with color coded dots on AFM topographs. Fibrillar aggregates after 6 h (Figure 4C) and 24 h (Figure 4E) exhibit a broad amide I band centered at 1664 cm^−1^ with a shoulder at 1634 cm^−1^ (Figure 4D,F), indicating that the key secondary structural element is still not beta sheet in nature. These fibril spectra are similar to the 1 h oligomers and to the disordered polymorphs observed during the equimolar aggregation. Interestingly, we do not observe any fibrils that have the beta sheet peak as the most prominent spectral signature. Fibrillar networks after 72 h (Figure 4G) showed a peak at 1664 cm^−1^, with a more pronounced shoulder at 1634 cm^−1^ (Figure 4H), indicating a relative increase in beta character, but not to the point where it is the dominating structural motif. Again, we do not find any fibril exhibiting prominent beta sheet character, which suggests that the polymorph with ordered beta structure is absent from the aggregation ensemble. Additionally, at all aggregation time points, the spectra are distinctly more homogeneous, which is in stark contrast with pure Aβ. To better quantify the above observations, we have used the ratio of the spectral intensity at 1666 cm^−1^ to that at 1636 cm^−1^ ($I_{1666}:I_{1636})$ as a metric reflective of the relative population of beta sheet and non-beta-sheet/disordered structures. Similar metrics have been previously used in IR microscopy to identify beta sheet content in amyloid plaques [33,34]. The ratio values were calculated for each spectrum shown in Figure 1, Figure 2, Figure 3 and Figure 4, and the mean ratio values are plotted as bar graph in Figure 5. We observed a higher value of the ratio for the 1:3 aggregates (yellow bars) compared to free Aβ (blue bars) at all time points, indicating that these aggregates contain more disordered secondary structure on average. In case of aggregation of Aβ with equimolar β-carotene, it can be clearly seen that the ratio consistently exhibits a lower value for the ordered polymorph (orange bars) in comparison to the disordered polymorph (gray bars), which suggests that the former has significantly more beta sheet content than the latter. The ratio values for the ordered polymorph are close to those of pure Aβ, which implies that their underlying secondary structure distribution is similar. These results clearly indicate that, at increased molar ratios of β-carotene, the aggregation ensemble is different, and there is only one fibril polymorph that exhibits non-beta-structure. It is possible that the ordered polymorph with beta sheets represents an aggregate species that does not or minimally associates with β-carotene, whereas the disordered polymorph without extensive beta structure represents a greater integration/association with the peptide. However, this is not possible to unequivocally determine in this study, since the infrared spectra of β-carotene does not have any bands in the fingerprint region that are unique and not found in proteins. Moreover, as discussed above, the AFM-IR signal from pure β-carotene is significantly weaker than the protein, which makes it more challenging to identify its spectral signatures. One strategy that can potentially be useful for spectroscopically isolating signatures of Aβ and β-carotene is isotope labeling. It is well known that amide bands exhibit a shift of ~30 cm^−1^ upon ^13^C substitution [35,36], which can be leveraged to avoid spectral overlap and identify individual vibrational signatures of Aβ and β-carotene. Alternatively, deuteration of β-carotene can also be explored. We aim to address this in future work. It is important to note, in this context, that the spectra shown in Figure 1, Figure 2, Figure 3 and Figure 4 are representative, and are not meant to indicate an exact statistical distribution. In fact, it is difficult to draw statistical interpretations regarding precise relative distributions of the structures identified in AFM-IR and, more generally, in any morphological characterization technique such as AFM or electron microscopy, since the entire ensemble is not measured. Hence, we have refrained from commenting on the relative abundance of the polymorphs in the structural ensemble and have chosen to interpret the results as indicative of the presence of one or more structures, or as one structure being more prevalent over the other, at best. The spectra reported herein suggest the presence of different polymorphs in the ensemble, but should not be viewed as exact indicators of relative abundance of the same. Taken together, the above findings nonetheless clearly suggest that the key role of β-carotene in modulating the aggregation pathway of Aβ is the promotion of fibrillar polymorphs with disordered, non-beta-structure, the relative abundance of which increases with molarity of β-carotene in the aggregation mixture. Furthermore, it also acts to reduce the structural heterogeneity between aggregates, essentially promoting a uniform structural ensemble where individual fibrils lack the structural ordering of stacked beta sheets, but this disordered structure is present in all fibrils.

The present study highlights the structural effects that can be induced by β-carotene on Aβ42 aggregates starting from the oligomeric stage. While previous measurements have identified that β-carotene modulates Aβ aggregation, the precise effect it has on the structure of the aggregates is difficult to discern without structural resolution at the single aggregate level. Earlier studies have shown similar results with Aβ40, where thioflavin T (ThT) assays indicated a slowing down of the aggregation kinetics in both 1:1 and 1:2 ratio aggregation mixture, instead of complete inhibition. It has also been demonstrated that Aβ40 fibrils become destabilized by the application of retinol, which is formed from provitamin A or β-carotene [26]. However, observation of reduced beta sheet character in presence of β-carotene cannot be unequivocally attributed to aggregation being arrested at the oligomeric/prefibrillar stage or to a significant change in the fibrillar structure. AFM-IR provides the opportunity to obtain both morphological and structural data simultaneously from the individual oligomers and fibrils, and our results clearly show that β-carotene does not completely inhibit the fibril formation. We have seen formation of fibrils from the oligomers for both 1:3 and 1:1 molar ratios of Aβ42 and β-carotene. A recent study with bexarotene, which is known to inhibit the Aβ42 aggregation, has also shown the formation of fibrils in its presence [37]; however, any significant alteration of the fibril secondary structure was not observed. In contrast, β-carotene transforms the structure of the aggregates starting at the oligomeric stage, leading to less heterogeneity between different aggregates at a given stage of aggregation and the formation fibrils with a distinct lack of beta sheet structure. To the best of our knowledge, this has never been demonstrated before and offers a new perspective into the modification of amyloid structure with carotenoids and related molecules. This work also highlights the advantage of spatially resolved nanoscale spectroscopic approaches such as AFM-IR over conventional ensemble averaged techniques such as FTIR. When the ensemble of aggregates is heterogeneous, as is expected to be the case for early stages of amyloid aggregation [29], AFM-IR allows for determination of the structure of individual aggregates, and, thus, mapping out the structural distribution of the ensemble. This is not easily determinable from a combination of FTIR and/or morphological characterization techniques such as electron microscopy/AFM. Studies have shown that AD patients have lower level of β-carotene and vitamin A in plasma. Moreover, higher levels of vitamin A are associated with better memory in elderly people [38]. Vitamin A deprivation has been shown to result in amyloid deposition in mouse brain [39]. More recently Raman microspectroscopy has identified the presence of carotenoids in amyloid plaques, which has been suggested to play an anti-inflammatory role to mitigate AD progression [24,25]. All these results emphasize the significant role of β-carotene and vitamin A in the pathogenesis of AD. The results reported herein on structural modification of Aβ42 aggregates by β-carotene highlights a potential pathway through which these types of molecules function and underscore the need of spatially resolved spectroscopic methods to unravel the molecular mechanism by which these natural products could be potentially used as alternate therapeutic strategies for AD.

## 3. Materials and Methods

### 3.1. Aggregation of Aβ42

Aβ42 (rPeptide, Watkinsville, GA, USA) was first treated with 1,1,1,3,3,3-hexafluoroisopropanol (HFIP) (Sigma-Aldrich, Co., St. Louis, MO, USA) to destroy any preformed oligomers. Then HFIP was completely evaporated under vacuum desiccator overnight. Aggregation of 100 μM Aβ42 was performed in 50 mM 2-(N-morpholino)ethanesulfonic acid (MES), 100 mM NaCl, and 0.5 mM EGTA (Sigma-Aldrich, Co., St. Louis, MO, USA), pH 6.5, with 1.5% DMSO (Sigma-Aldrich, Co., St. Louis, MO, USA) at 37 °C without any agitation. 

### 3.2. Aggregation of Aβ42 with β-Carotene

A 20 mM stock solution of β-carotene (Sigma-Aldrich, Co., St. Louis, MO, USA) was prepared in DMSO. Aggregation of Aβ42 in presence of β-carotene was performed in two molar ratios of 1:1 and 1:3, with Aβ42 concentration of 100 μM in the aggregation mixture. Aggregation condition was kept similar to that of free Aβ42. 

### 3.3. Sample Preparation for AFM-IR Experiment

Samples were prepared by taking out aliquots from the aggregation mixture at 1 h, 6 h, 24 h, and 72 h and depositing half-diluted solutions onto ultraflat gold substrates (Platypus Technologies, Madison, WI, USA). The droplet was incubated on the gold substrate for 5 min and then rinsed with ~100 μL of Milli-Q water. The sample was dried under a gentle stream of nitrogen gas and kept inside a vacuum desiccator until imaging.

### 3.4. AFM-IR Experiment

AFM-IR experiments were carried out with a Bruker NanoIR3 instrument (Bruker Corporation, Santa Barbara, CA, USA) equipped with a mid-IR quantum cascade laser (MIRcat, Daylight solutions, San Diego, CA, USA). Experiments were performed at room temperature, and relative humidity inside the instrument was kept low by continuous purging with dry air. Both AFM imaging and IR data collection was performed in tapping mode with cantilevers having a resonance frequency of 75 ± 15 kHz and a spring constant of 1–7 N/m. The AFM scan rate was kept at 1.0 Hz. First, a high-resolution AFM image of the sample was recorded having multiple oligomers/fibrils in the scan area. The AFM tip was placed on individual oligomer/fibril, chosen at random, to obtain the IR spectra. Three different areas of sample were scanned where multiple oligomer/fibrils were probed for each time point to avoid any biasness or under sampling. Typically, 30–50 spectra over the three areas were acquired on oligomer/fibril features for each time point. For oligomers, a lesser number of spectra were acquired compared to fibrils, owing to lesser abundance. IR spectral resolution was 2 cm^−1^. A total of 128 coadditions at each point and 16 co-averages for each spectrum were applied.

### 3.5. Data Analysis

AFM images were processed by Gwyddion software. IR data were analyzed using MATLAB software by applying (3, 7) Savitzky–Golay filter and a baseline correction for each spectrum.

## Data Availability

All data needed to evaluate the conclusions of the paper are provided in the paper or as Supplementary Information. The datasets generated and/or analyzed in this study are available from the corresponding author upon reasonable request.

## Supplementary Materials

The supporting information can be downloaded at: https://www.mdpi.com/article/10.3390/ijms24065175/s1.
