# Supplementary material for: β-Carotene, a Potent Amyloid Aggregation Inhibitor, Promotes Disordered Aβ Fibrillar Structure
Source: Int J Mol Sci. 2023 Mar 8;24(6):5175. doi: 10.3390/ijms24065175 (PMC10049578; doi:10.3390/ijms24065175)
Supplement: Supplementary file 1 [file ijms-24-05175-s001.zip › ijms-2228159-supplementary.pdf]

Supporting Information

β-Carotene, a Potent Amyloid Aggregation Inhibitor, Promotes Disordered Aβ Fibrillar Structure

Siddhartha Banerjee †, Divya Baghel †, Ana Pacheco de Oliveira and Ayanjeet Ghosh *

Department of Chemistry and Biochemistry, The University of Alabama, 1007E Shelby Hall, Tuscaloosa, AL 35487, USA

* Correspondence: ayanjeet.ghosh@ua.edu

† These authors contributed equally to this work.

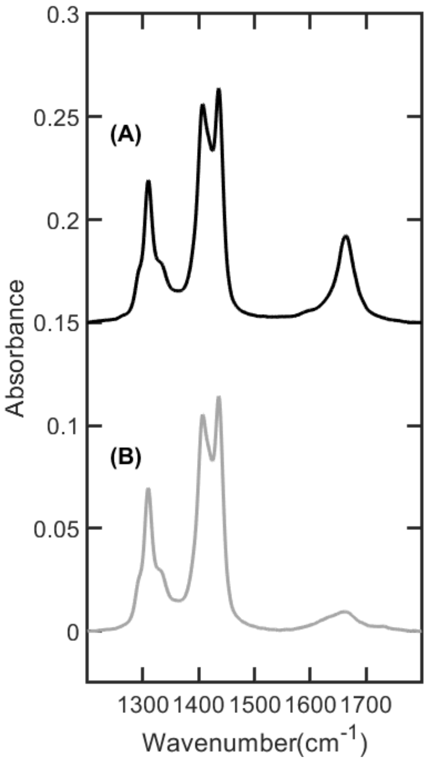

Figure S1. FTIR spectra of (A) 1 mM Aβ42 stock and (B) pure β-carotene in DMSO. The spectra have been vertically offset for clarity. The relative intensity of β-carotene in the amide I region (1600-1700cm^{-1}) is significantly weaker than the peptide. The intense bands between 1300-1450cm^{-1} mainly arise from the solvent DMSO.

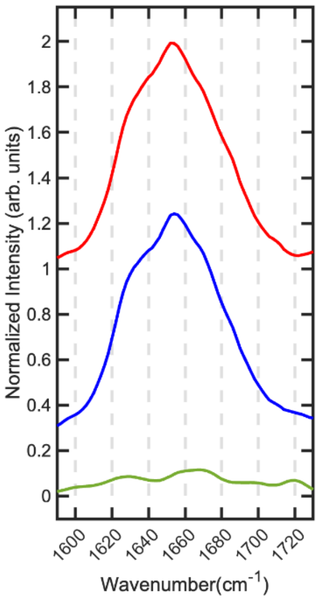

Figure S2. Comparison of relative AFM-IR responses of β-carotene and Aβ42 in the amide region. β-carotene presents a broad, featureless band (green) and much weaker compared to typical protein peak (blue). Red spectrum shows the protein amide I band after subtraction of β-carotene contribution.

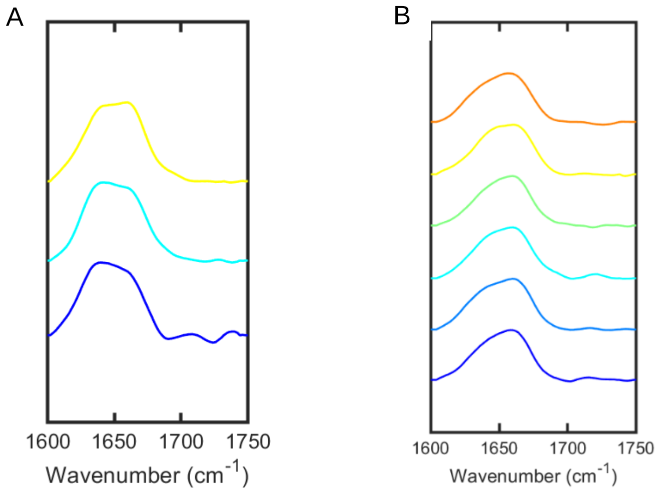

Figure S3. (A) β-sheet characteristic amide I bands those have been rarely observed in Aβ42 aggregation in presence of equimolar β-carotene. (B) Spectra recorded on protofibrilar aggregates.
